# Mutations in SARS-CoV-2 structural proteins: a global analysis

**DOI:** 10.1186/s12985-022-01951-7

**Published:** 2022-12-18

**Authors:** Mohammad Abavisani, Karim Rahimian, Bahar Mahdavi, Samaneh Tokhanbigli, Mahsa Mollapour Siasakht, Amin Farhadi, Mansoor Kodori, Mohammadamin Mahmanzar, Zahra Meshkat

**Affiliations:** 1 Student Research Committee, Mashhad University of Medical Sciences, Mashhad, Iran; 2 Department of Microbiology and Virology, School of Medicine, Faculty of Medicine, Mashhad University of Medical Sciences, Mashhad, Iran; 3 Institute of Biochemistry and Biophysics (IBB), University of Tehran, Tehran, Iran; 4 Department of Molecular Biotechnology, Cell Science Research Center, Royan Institute for Biotechnology, ACECR, Isfahan, Iran; 5 Department of Molecular and Cellular Sciences, Faculty of Advanced Sciences and Technology, Pharmaceutical Sciences Branch, Islamic Azad University, Tehran, Iran; 6 Department of Biochemistry, Erasmus University Medical Center, P.O. Box 2040, 3000 CA Rotterdam, The Netherlands; 7 Department of Biology, Payame Noor University, Tehran, Iran; 8 Non Communicable Diseases Research Center, Bam University of Medical Sciences, Bam, Iran; 9 Department of Bioinformatics, Kish International Campus University of Tehran, Kish, Iran

**Keywords:** COVID-19, Evolutionary analysis, Genome-wide mutations, Mutations, SARS-CoV-2

## Abstract

**Background:**

Emergence of new variants mainly variants of concerns (VOC) is caused by mutations in main structural proteins of severe acute respiratory syndrome coronavirus 2 (SARS-CoV-2). Therefore, we aimed to investigate the mutations among structural proteins of SARS-CoV-2 globally.

**Methods:**

We analyzed samples of amino-acid sequences (AASs) for envelope (E), membrane (M), nucleocapsid (N), and spike (S) proteins from the declaration of the coronavirus 2019 (COVID-19) as pandemic to January 2022. The presence and location of mutations were then investigated by aligning the sequences to the reference sequence and categorizing them based on frequency and continent. Finally, the related human genes with the viral structural genes were discovered, and their interactions were reported.

**Results:**

The results indicated that the most relative mutations among the E, M, N, and S AASs occurred in the regions of 7 to 14, 66 to 88, 164 to 205, and 508 to 635 AAs, respectively. The most frequent mutations in E, M, N, and S proteins were T9I, I82T, R203M/R203K, and D614G. D614G was the most frequent mutation in all six geographical areas. Following D614G, L18F, A222V, E484K, and N501Y, respectively, were ranked as the most frequent mutations in S protein globally. Besides, A-kinase Anchoring Protein 8 Like (AKAP8L) was shown as the linkage unit between M, E, and E cluster genes.

**Conclusion:**

Screening the structural protein mutations can help scientists introduce better drug and vaccine development strategies.

**Supplementary Information:**

The online version contains supplementary material available at 10.1186/s12985-022-01951-7.

## Background

Coronavirus disease 2019 (COVID-19) was declared a pandemic on March 11, 2020. The disease is caused by severe acute respiratory syndrome coronavirus 2 (SARS-CoV-2), a single-stranded, positive-sense RNA virus with a genome size of 29,903 nucleotides [1]. The infection and mortality rates of the disease vary among different countries, and the precise factors influencing the variability are unknown [2]. Despite the proofreading mechanisms of the virus, mutation rates of coronaviruses are between 10^− 5^ and 10^− 3^ substitutions per nucleotide site per cell infection (s/n/c); therefore, several mutations have been detected by wide-range sequencing [3, 4]. The genome of SARS-CoV-2 contains 12 open reading frames (ORF) that encode 22 nonstructural proteins and 4 structural proteins, including envelope (E), membrane (M), nucleocapsid (N) and spike (S) proteins [5].

The S protein, which plays a crucial role in recognition by human host cell surface receptor angiotensin-converting enzyme 2 (ACE-2), consists of an N-terminal S1 subunit and a C-terminal S2 subunit. The occurrence of a point mutation in the S1 subunit named D614G mutation has resulted in the dominant variant of SARS-CoV-2. This mutation is associated with a higher viral load, increased fitness, and enhanced virus infectivity, thereby leading to disease emergence at a younger age [3, 6–9]. In fact, emergent mutations may appear as a new variant of concern (VOC), leading to an increased potential in binding affinity to human receptors and higher infectivity and mortality power than previous variants. The B.1.617.2 (Delta) variant is an example of such demonstration, which has higher transmissibility and is resistant to neutralization due to the accumulation of mutations in the S protein [10]. The B.1.1.529 (Omicron) variant is the fifth and, up to now, the last VOC possessing about 40 mutations containing one mutation in E protein, three mutations in M protein, six mutations in N protein, and more than thirty changes in S protein. It was discovered in South Africa in November 2021, with increased infectivity and a higher potential for stimulating the immune response due to many mutations [11]. These results emphasize the need to better understand how mutation patterns affect COVID-19 prevalence, infectivity, and mortality rate across countries.

At present, vaccination is the most practical strategy for combating COVID-19. Most designed vaccines inhibit viral pathogenesis by targeting the S protein [12, 13]. Hence, investigating the effect of the mutations is vital in determining the quality of vaccinations. On the other hand, some of the therapeutic strategies are based on the interaction between drugs and S proteins [14–16]. Other structural proteins are also considered attractive targets for synthetic drugs and vaccine formulations [17, 18]. Consequently, identifying the geographical distributions and evolutionary trends of the structural proteins of SARS-CoV-2 triggers the targeted approach in epidemiological research and molecular design of vaccines and drugs.

In the current study, we aimed to discover the frequencies of mutations among structural proteins of SARS-CoV-2 globally and in different continents separately. Then, we addressed the evolutionary trends of the mutation, taking into account their characteristics from the beginning of the pandemic to January 2022, followed by investigating the interactions between human genes and structural proteins of SARS-CoV-2.

## Materials and methods

### Sequence retrieval

**Fig. 1:**
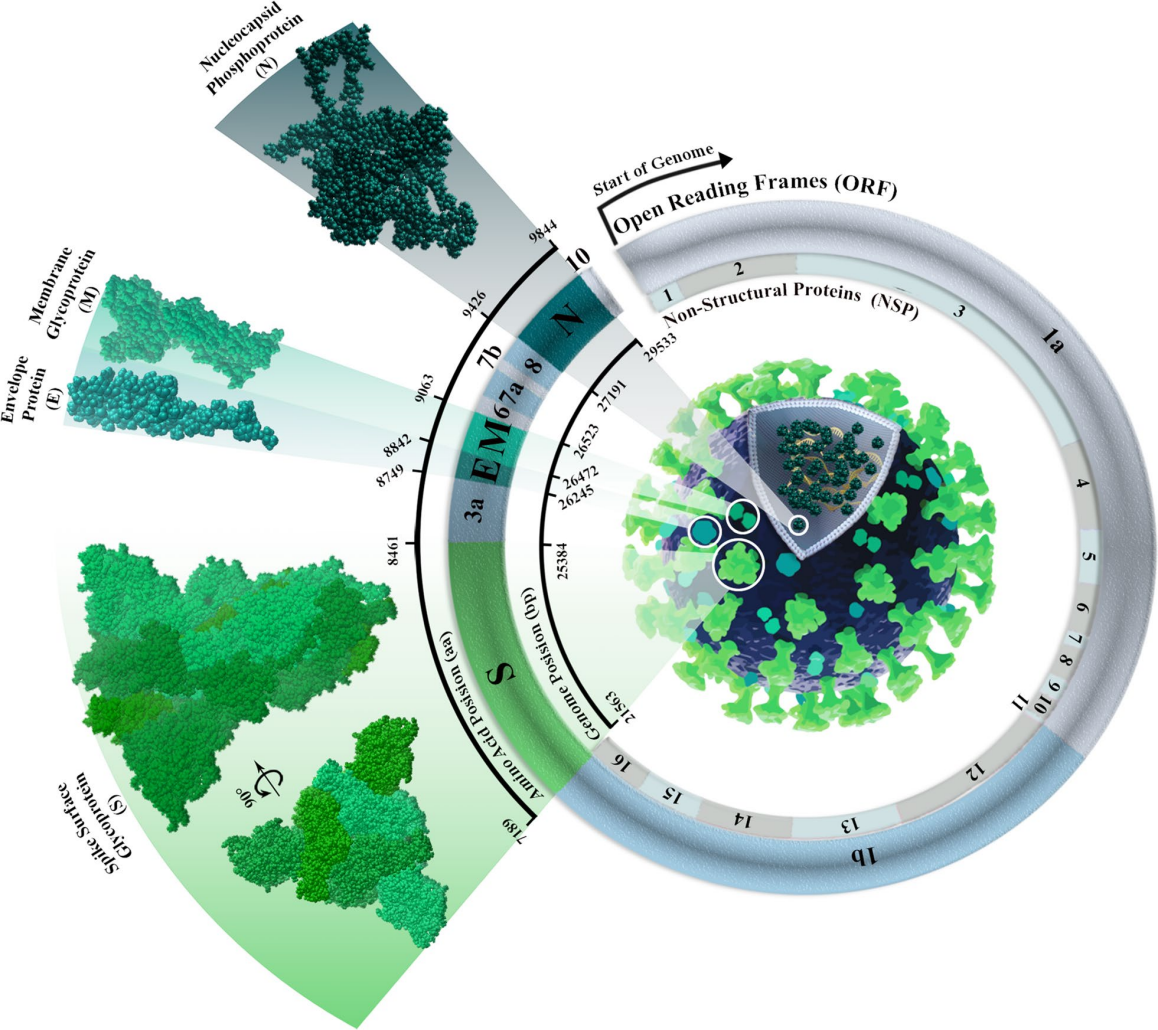
The genomic positions of four structural proteins in SARS-CoV-2. E protein includes 26,245 to 26,472, and subsequently, the positions of the M, N, and S proteins are 26,523 to 27,191, 28,274 to 29,533, and 21,563 to 25,384, respectively

The current study was carried out by analyzing whole data from AASs of four structural proteins of SARS-CoV-2. All AASs were evaluated in comparison to the reference sequence. We used the AAS of the Wuhan-2019 virus with access number ‘EPI ISL 402124’ as the reference sequence. GISAID (https://www.gisaid.org) was used to extract AASs from the E (with 75 AA owned by 6,524,654 samples), M (with 222 AA owned by 6,524,705 samples), N (with 419 AA owned by 6,523,549 samples), and S (with 1273 AA owned by 6,603,870 samples) regions from January 2020 to January 2022. Erasmus Medical Center granted access to this database (https://www.gisaid.org/collaborations/collaboration-on-h5-antigenic-cartography) [19–21]. The genomic position of the evaluated amino acids ranges from 26,245 to 26,472 for the E protein, 26,523 to 27,191 for the M protein, 28,274 to 29,533 for the N protein, and 21,563 to 25,384 for the S protein (Fig. 1).

### SARS-CoV-2 sequences analyses

Python 3.8.0 software was used to read the FASTA files, extract the four mentioned structural proteins, and align and analyze the SARS-CoV-2 sequences to detect the presence of any mutations. Each difference between the sample and reference sequences was interpreted as a mutation, and the location as well as the substituted AA, were reported. Non-human samples, samples with a different number of AAs than the reference, and samples with unspecified AAs were excluded from each structural protein. The entire process was optimized using the ‘Numpy’ and ‘Pandas’ libraries.

The algorithm utilized for detecting mutations is as follows:

For refitem, seqitem in zip (refseq, seq).

If (refitem! =seqitem).

Report a new mutant.

Where ‘refseq,’ and ‘seq’ refer to the reference and sample sequences, respectively.

The continent name and geographical coordinates for each sample were obtained and reported using the pycountry-convert 0.5.8 software and the ‘Titlecase’ library in Python to depict the global prevalence maps of mutations. Finally, global maps were created with ‘Geobubble’ package of Matlab 2021.

### Data normalization and statistical analysis

Data normalization was performed using R 4.0.3 and Microsoft Power BI. The normalized frequency of each studied region was obtained for a more accurate comparison of the data attributed to each continent. Accordingly, the number of mutations was divided by the number of comparable sequences on that continent in equal proportions.

### Identification of related human genes and interactions

The Human Protein Atlas database (https://www.proteinatlas.org/humanproteome/sars-cov-2) was used to find human genes related to E, M, N, and S genes in SARS-CoV-2. STRING ver.11.5, with an average local clustering coefficient of 0.527, was used to determine the interaction between target genes. An adjacent matrix was created and imported into Cytoscape version 3.8 to visualize the PPI network. In addition, the cytoHubba package was used to perform node ranking analysis to identify the hub genes.

## Results

### Mutation quantities among geographical areas

At the start, we decided to find the mutations to understand mutation incidence rates and statistically identify essential mutations. A total of 6,394,483, 6,177,403, 5,841,477, and 895,738 E, M, N, and S sequences were studied, respectively.

**Fig. 2:**
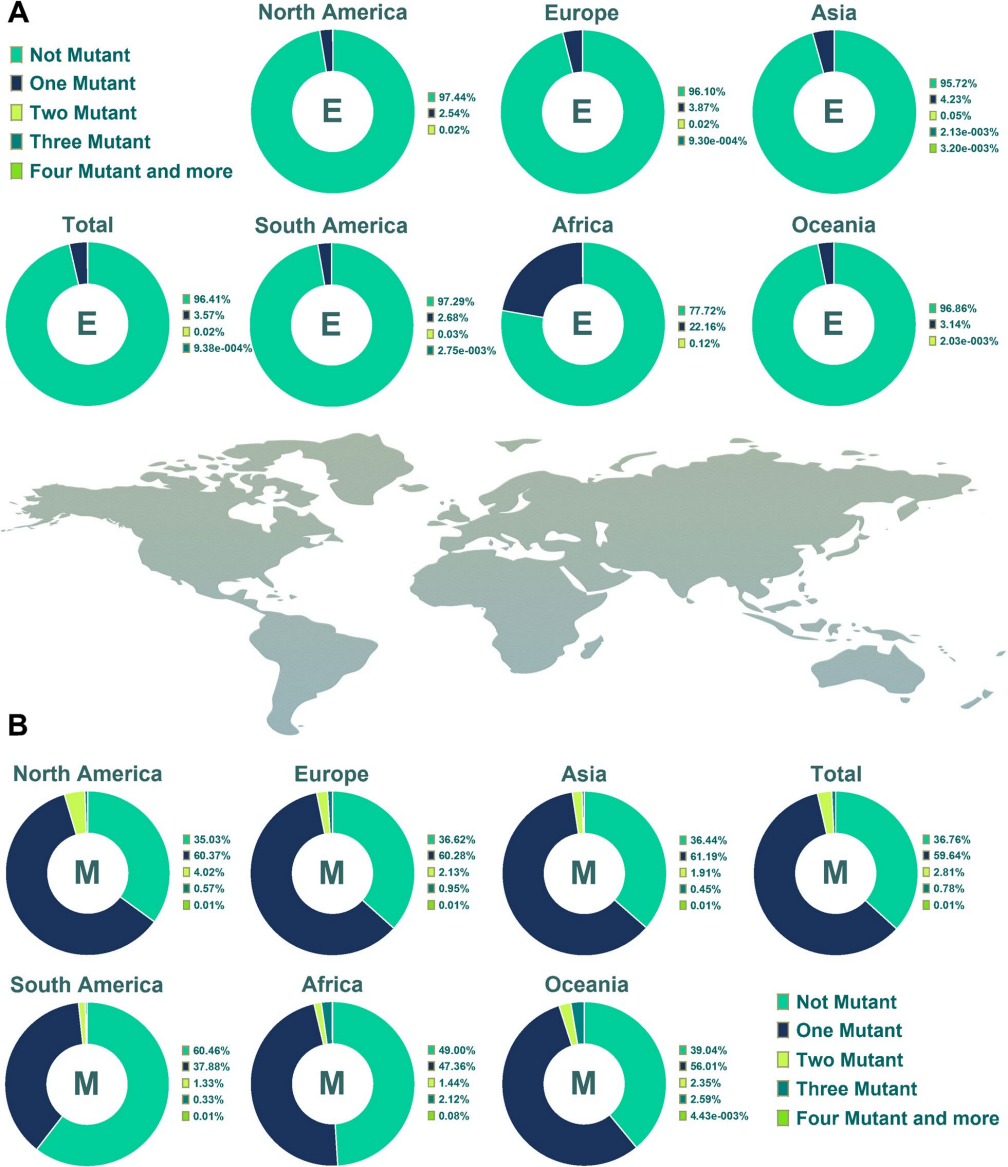
Pie chart plot of the number of mutations among E and M proteins of SARS-CoV-2 up to January 2022. The incidence rate of one, two, three, four, and more mutations, in addition to the rate of lack of any mutation among these proteins, has been displayed in the clusters A and B

According to the obtained data, 96.40% of the E amino acid (AA) sequences (AASs) exhibited no mutation (Fig. 2A). These characteristics were 36.76%, 2.20%, and 2.11% for M, N, and S AASs, respectively. Furthermore, in the same order, 3.56%, 59.64%, 5.68%, and 26.86% had one mutation. Two mutations were found in 0.02%, 2.80%, 7.11%, and 26.15% of E, M, N, and S AASs, respectively.

The data from the E protein demonstrated that 77.72% of AASs in Africa and 95.72% of Asia ASSs did not display any mutation. This exhibition in Europe, North America, South America and Oceania has been observed in 96.10%, 97.44%, 97.30% and 96.86% of AASs, respectively. In comparison, 49% of Africa ASSs did not exhibit any mutation for the M protein (Fig. 2B). Such a feature for M protein was concluded by 36.44%, 36.62%, 35.03%, 60.45% and 39.04% in Asia, Europe, North America, South America and Oceania, successively. One mutation of M protein was shown in 47.35%, 61.19%, 60.27%, 60.36%, 37.87% and 56% in Asia, Europe, North America, South America and Oceania ASSs, respectively. 1.43%, 1.9% and 2.13% of Africa, Asia and Europe ASSs illustrated two mutations in M ASSs, same as the 4.01%, 1.33% and 2.35% of ASSs in North America, South America and Oceania, successively.

**Fig. 3:**
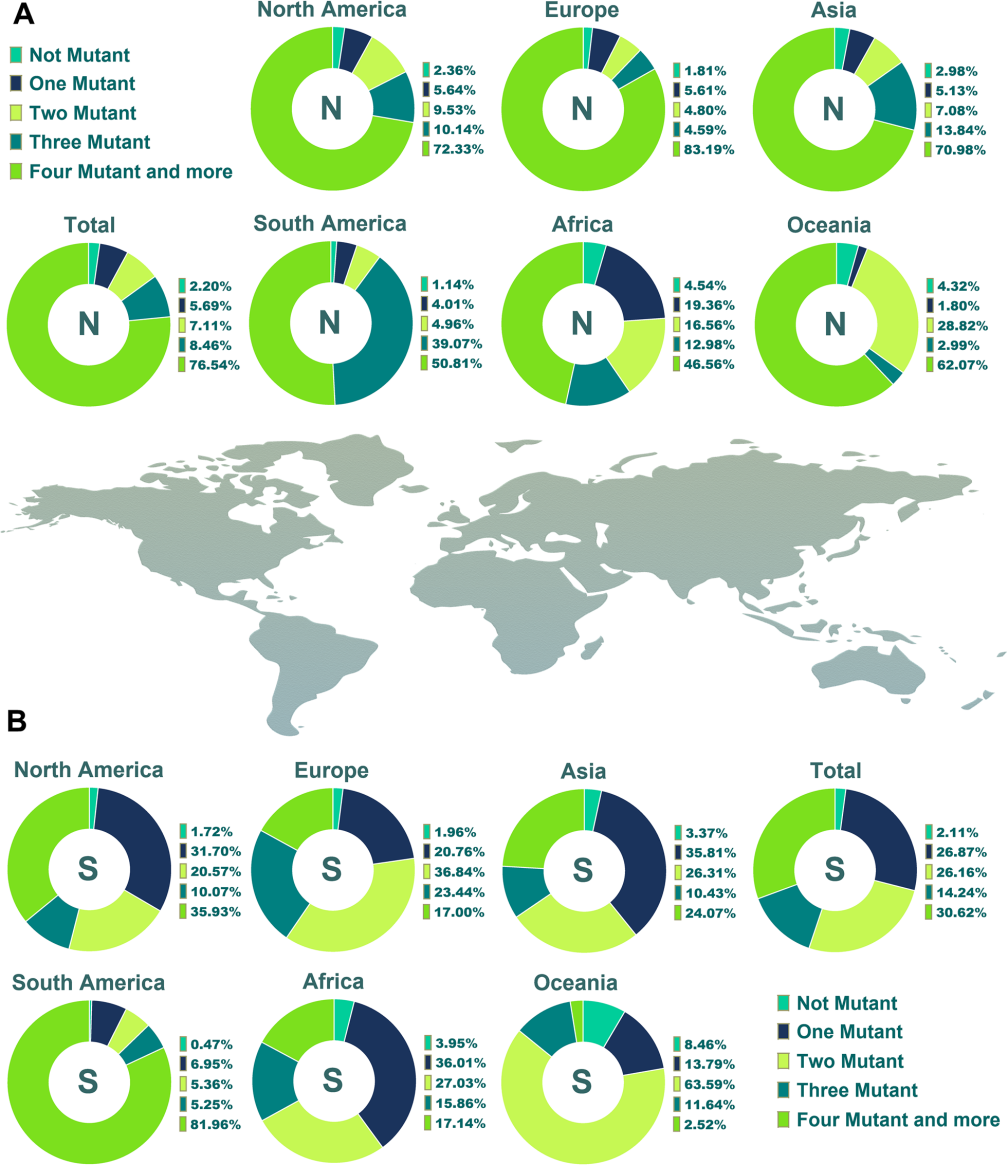
Pie chart plot of the number of mutations among N and S proteins of SARS-CoV-2 up to January 2022. The frequency of one, two, three, four, and more mutations, as well as the absence of any mutation, has been shown in the A and B clusters

Furthermore, 4.53% of the African samples did not have any N protein mutation (Fig. 3A). Also, 2.98%, 1.80%, 2.36%, 1.14% and 4.31% of N AASs in Asia, Europe, North America, South America and Oceania were without mutation occurrence, respectively. In contrast to Africa, which displayed 19.35% with one mutation, the one-mutation incidence rates in the other five areas were noticeably lower, and except for Oceania, other areas displayed almost similar one-mutation incidence rates. The percentages of N ASSs with two mutations in Oceania and Africa were higher than in other areas. This demonstration in Oceania and Africa ASSs was 28.81% and 16.55%, respectively but in Asia, Europe, North America and South America this trend was 7.07%, 4.8%, 9.53% and 4.95%, successively. Concerning the S protein, it has resulted that in South America, only 0.46% of AASs did not display any mutation and about 82% demonstrated four and more mutations (Fig. 3B). Oceania’s no-mutation incidence rate for this protein was 8.45%, the highest no-mutation incidence rate. The one mutation incidence rate among S AASs has been demonstrated as 36.01%, 35.81%, 20.75%, 31.70%, 6.95% and 13.78% in the ASSs of Africa, Asia, Europe, North America, South America and Oceania, respectively. 81.96%, 35.92%, 24.07%, 17.14%, 16.99% and 2.52% of S AASs displayed four and more mutations among South America, North America, Asia, Africa, Europe and Oceania ASSs, respectively. The prevalence of AASs with one mutation in Africa, Asia and Europe was higher than in other types of achieved data. Besides, the most prevalent mutations in Oceania and the Americas were two and more than three, respectively.

**Fig. 4:**
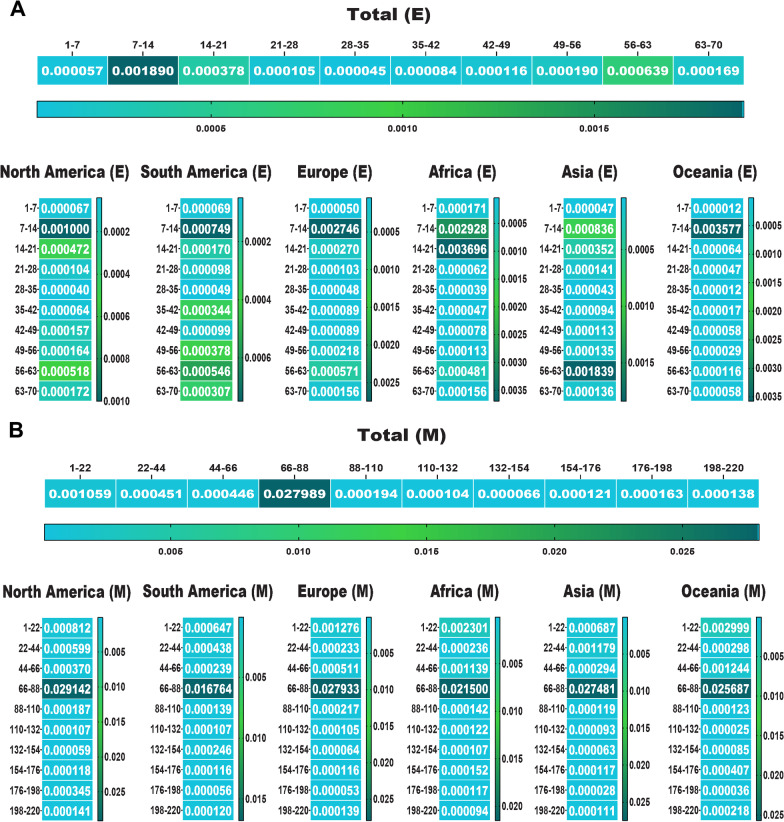
The heat map of mutations among E and M proteins of SARS-CoV-2 as of January 2022. These indicate the rate of mutation per 100 amino acids. The highest frequency rate of mutations among the E and M AASs occurred in the regions of 7 to 14 AA, and 66 to 88 AA, respectively

**Fig. 5:**
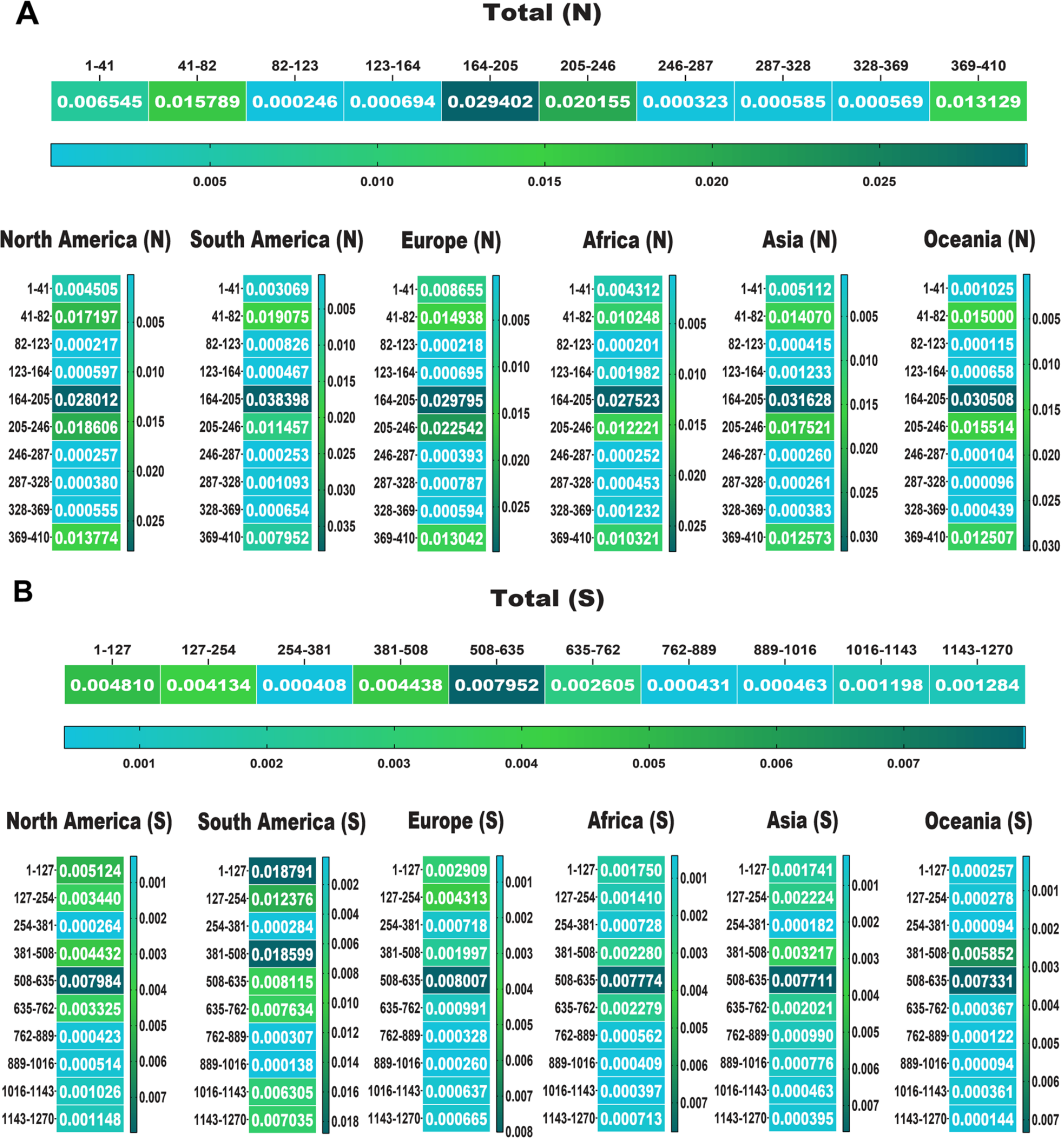
The heat map of mutations among the N and S proteins of SARS-CoV-2 as of January 2022. The regions with the highest frequency of mutations among the N and S AASs were 164 to 205 AA and 508 to 635 AA, respectively

In the following, we drew a heat map for mutations to detect their frequency in total and among each area. Data displayed the most mutations relative to the total AASs among the E, M, N and S AASs occurred in the regions of 7 to 14 AA (0.0018 frequency), 66 to 88 AA (0.0279 frequency), 164 to 205 AA (0.0294 frequency) and 508 to 635 AA (0.0079 frequency), respectively (Figs. 4, 5). The second highest mutations frequency in the E, M, N and S AASs arose in the regions of 56 to 63 AA (0.0006 frequency), 1 to 22 AA (0.0010 frequency), 205 to 246 AA (0.0201 frequency) and 1 to 127 AA (0.0048 frequency), respectively.

### The characteristics of mutations based on geographical areas

**Fig. 6:**
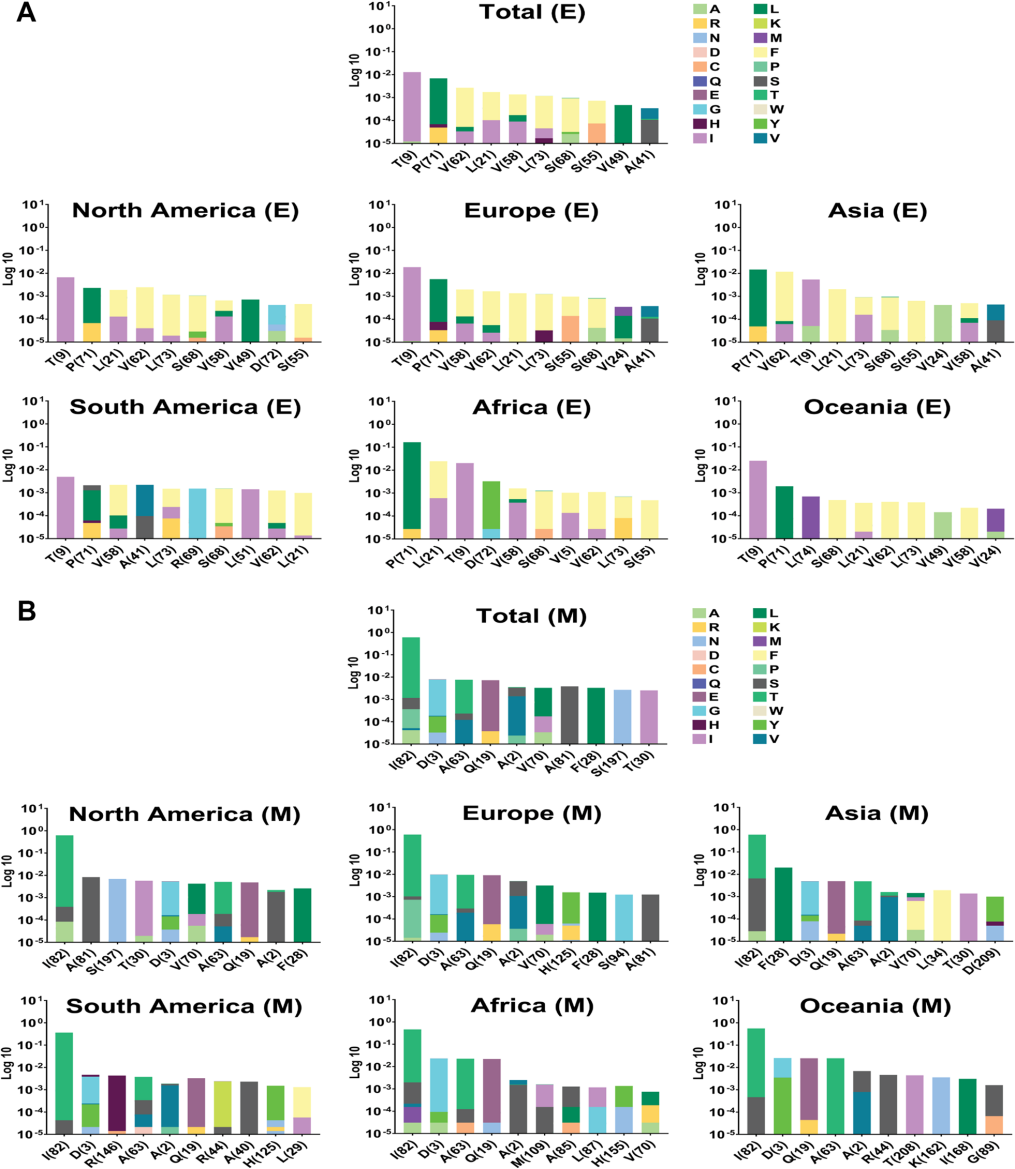
Top ten mutations among E and M with the highest frequency worldwide and geographic areas. The positions of altered and substituted AAs are shown differently based on the frequency percentage of the substituted AA. For better data representation, the data is represented by a logarithm based on 10. In total, T9I, P71L, V62F, L21F/L21V, V58F, L73F, S68F, S55F, V49L, and A41V were displayed as the ten mutations with the highest frequency rate of mutations for E AASs. I82T, D3G, A63T, Q19E, A2S, V70L, A81S, F28L, S197N, and T30I are the top ten frequent mutations among M AASs globally

The locations of mutations in the protein structure and their frequency were considered in the following step to identify more dimensions of mutations. As shown in Fig. 6A, T9I (0.0128) has the highest frequency of mutations in the E protein, followed by P71L (0.0068), V62F (0.0066), L21F/V (0.0017/0.0003), and V58F (0.0013). Accordingly, T9I was the most frequent mutation in Europe, Oceania, North America and South America, with 0.0187, 0.0249, 0.0066 and 0.0049 frequency rates, respectively. Nevertheless, P71L was the most frequent mutation in Africa and Asia, with frequency rates of 0.1643 and 0.0146, respectively. V62F is one of the first ten frequent mutations in Asia (0.0118 frequency), Europe (0.0016 frequency) and North America (0.0024 frequency), in contrast to Africa (0.0011 frequency), Oceania (0.0004 frequency) and South America (0.0012 frequency) which this mutation was as eighth, sixth and ninth, respectively.

Regarding the M protein, the analysis showed I82T (0.6015 frequency), D3G (0.0077 frequency), A63T (0.0073 frequency), Q19E (0.0072 frequency) and A2S (0.0033 frequency) are the first five mutations with the highest frequency, respectively (Fig. 6B). I82T was the most frequent mutation in all six areas. This situation differs from the D3G mutation, which was not the second most frequent mutation in Asia and North America. In these areas, F28L (0.020 frequency) and A81S (0.0083 frequency) were in the second position of frequent mutations, respectively. A63T was the third most frequent mutation in Africa and Europe, with 0.0224 and 0.0091 frequencies, respectively. On the other hand, the third most frequent mutation in Asia, Oceania, North America and South America were D3G (0.0048 frequency), Q19E (0.0259 frequency), S197N (0.0069 frequency) and R164H (0.004 frequency), respectively.

**Fig. 7:**
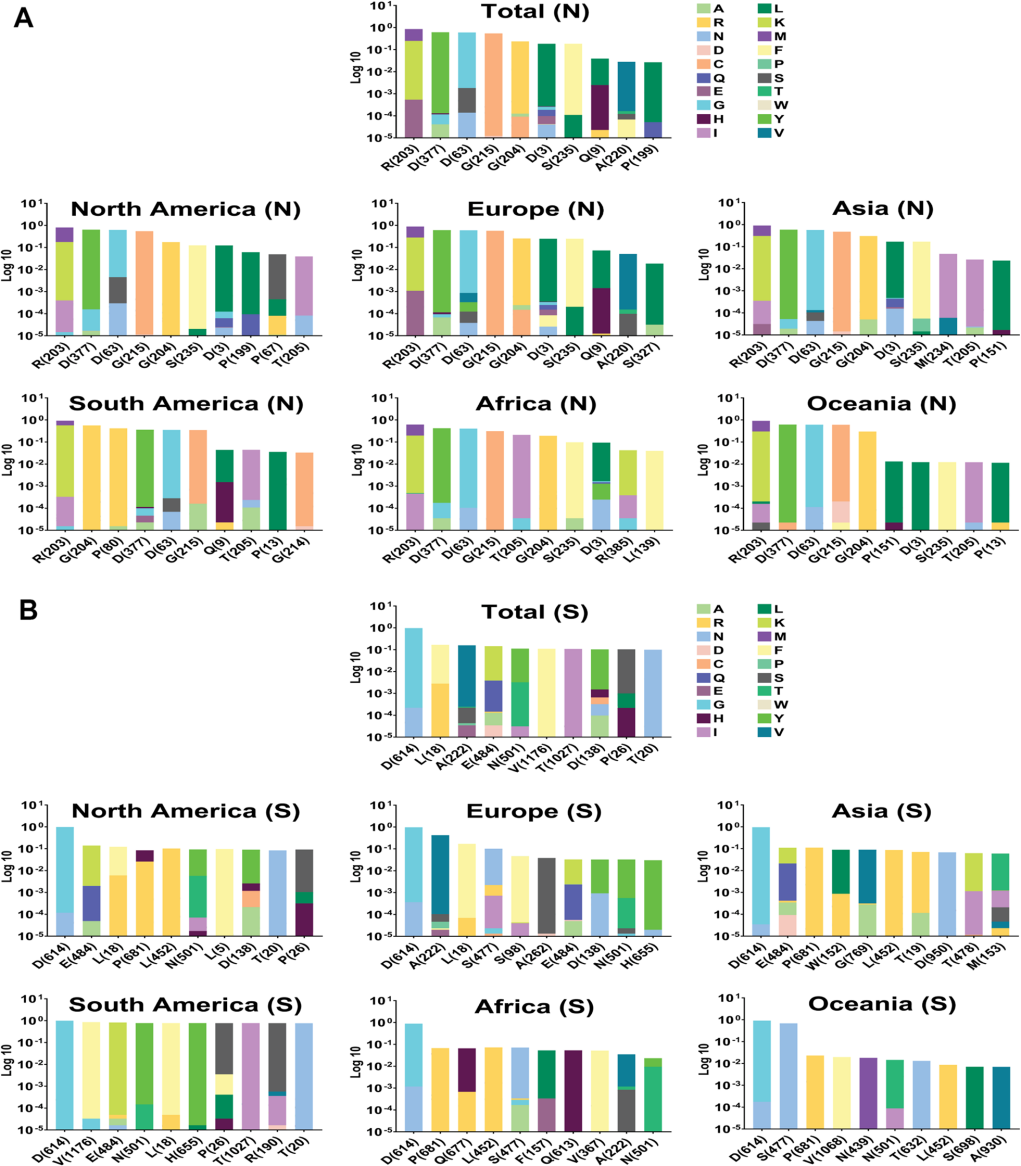
Top ten mutations among N and S with the highest frequency worldwide and geographic areas. R203M/R203K, D377Y, D63G, G215C, G204R/G204P, D3L, S235F, Q9L, A220V, and P199L rank first to tenth in frequency for M AASs in the entire world, and the top ten frequent mutations for S AASs were also concluded to be D614G, L18F, A222V, E484K, N501Y, V1176F, T1027I, D138Y, P26S, and T20N globally

Analysis of N AASs data illustrated that the R203M/R203K with 0.6084/0.2489 frequencies was at the first position of frequent mutations (Fig. 7A). Globally, D377Y (0.6134 frequency) mutation is the second, D63G (0.6002 frequency) the third, G215C (0.5479 frequency) the fourth and G204R/G204P (0.2352/0.0134 frequencies) the fifth mutation. In all continents except South America, up to the fourth position of frequent mutations were similar to the global results. Analysis of data from South America resulted in a different arrangement. The frequency of the R203M mutation was higher than the R203K mutation in all continents except South America. The R203M/R203K frequencies in Africa were 0.4195/0.1965, in Asia were 0.6033/0.3052, in Europe were 0.6074/0.2826, in North America were 0.6310/0.1776 and in Oceania were 0.6143/0.3008. However, in South America, the R203M/R203K frequencies were 0.3570/0.5700. A further dimension of differences between South America and other areas is the positions of second and third frequent mutations. G204R (0.5685 frequency) and P80R (0.4184 frequency) rank second and third mutations in South America.

**Fig. 8:**
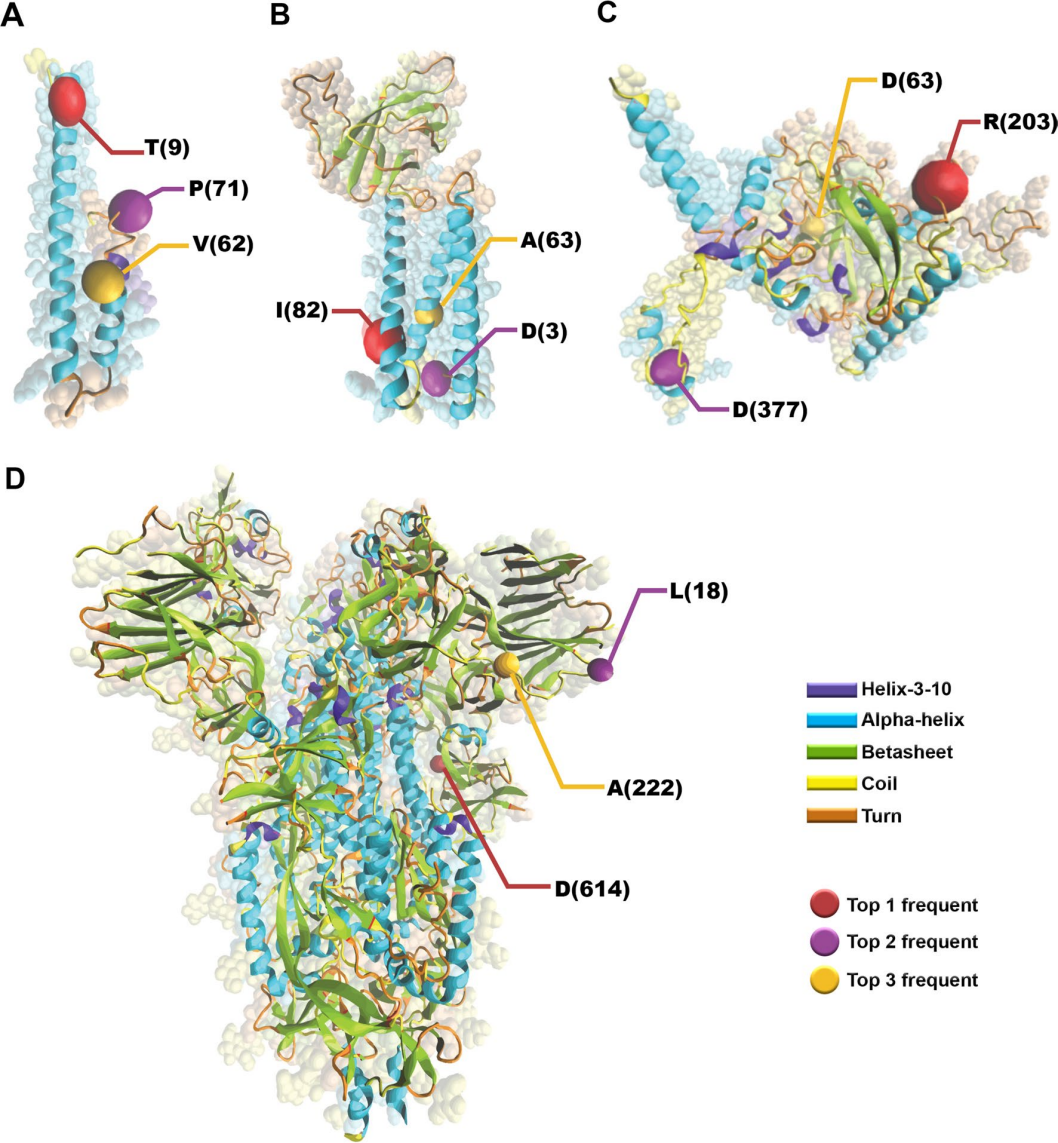
The locations of the top three frequent mutations occurred in E protein (**A**), M protein (**B**), N protein (**C**), and S protein (**D**). The mutations are among the top frequent mutations in the total

The pattern of mutation frequency for S AASs displayed that D614G, with 0.9756 frequency worldwide, achieved first place among frequent mutations. In the following, L18F (0.1680 frequency), A222V (0.1579 frequency), E484K (0.1454 frequency) and N501Y (0.1120 frequency) rank second to fifth frequent mutations (Fig. 7B). The first frequent mutation in S AASs was identical in all six geographical areas; however, the frequencies differed between them. The frequency of D614G in Africa, Asia, Europe, North America, South America and Oceania was 0.8884, 0.9579, 0.9743, 0.9835, 0.9959 and 0.9047, respectively. In Africa, P681R/P681H mutations (0.0674/0.0396 frequencies) rank second, Q677H/Q677K mutations (0.0661/0.0163 frequencies) rank third, L452R mutation (0.0728 frequency) ranks fourth and S477N mutation (0.0712) ranks fifth. In addition to the point that the second mutation in Asia attributed to E484K/E484Q (0.1112/0.0211 frequencies), P681R/P681H (0.1127/0.0150 frequencies), W152L (0.0897 frequency) and G769V (0.0903 frequency) are the third to fifth mutations in S AASs. In Europe, A222V (0.4217 frequency) is the second most frequent mutation and L18F (0.1702 frequency), S477N (0.0999 frequency) and S98F (0.0466 frequency) are the third to fifth, respectively. Among the mutations that occurred in S AASs from North America, E484K (0.1393 frequency) ranks second, L18F (0.1227 frequency) ranks third, P681H/P681R (0.0849/0.0268 frequencies) ranks fourth and L452R (0.1021) ranks fifth which are different in the types in frequent mutations from South America. In South America, V1176F with 0.8592 frequency, E484K with 0.8268 frequency, N501Y with 0.7745 frequency and L18F with 0.7742 frequency are the frequent mutations, respectively. S477N (0.6864 frequency), P681R (0.0229 frequency), V1068F (0.0196 frequency) and N439K (0.0179 frequency) are the frequent mutation following the D614G mutation in Oceania, respectively. Additional data have been listed in Additional files 1, 2, 3 and 4. As a conclusive visualization, the locations of top three frequent mutations were determined in Fig. 8.

### Evolutionary trends of emergence and distribution of top ten mutations concerning the time and geographical areas

**Fig. 9:**
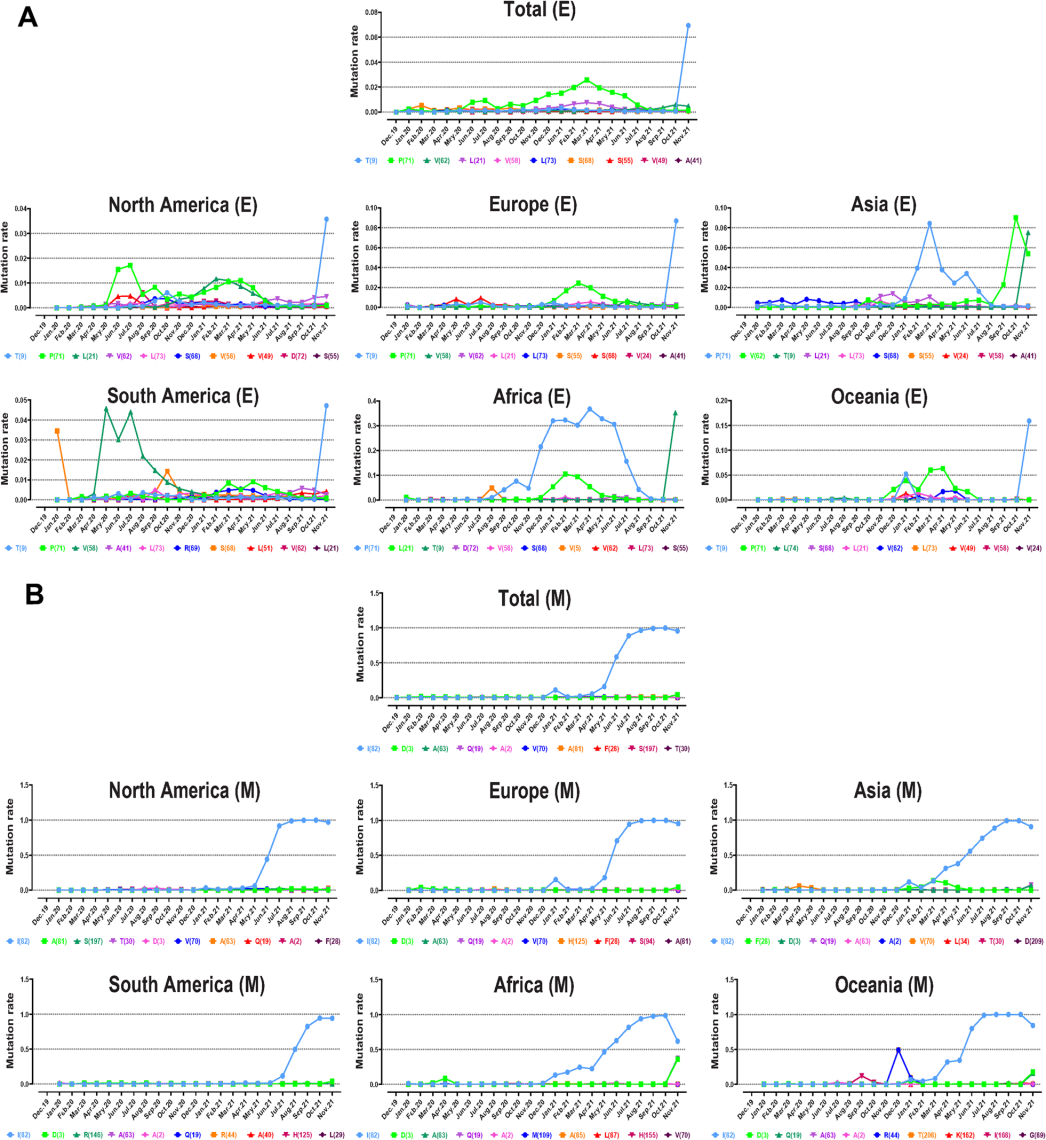
Timeline for report of mutations and evolutionary trends of the top ten high-rate mutations in E and M proteins based on the regions of North America, South America, Europe, Asia, Oceania, Africa, and globally

**Fig. 10:**
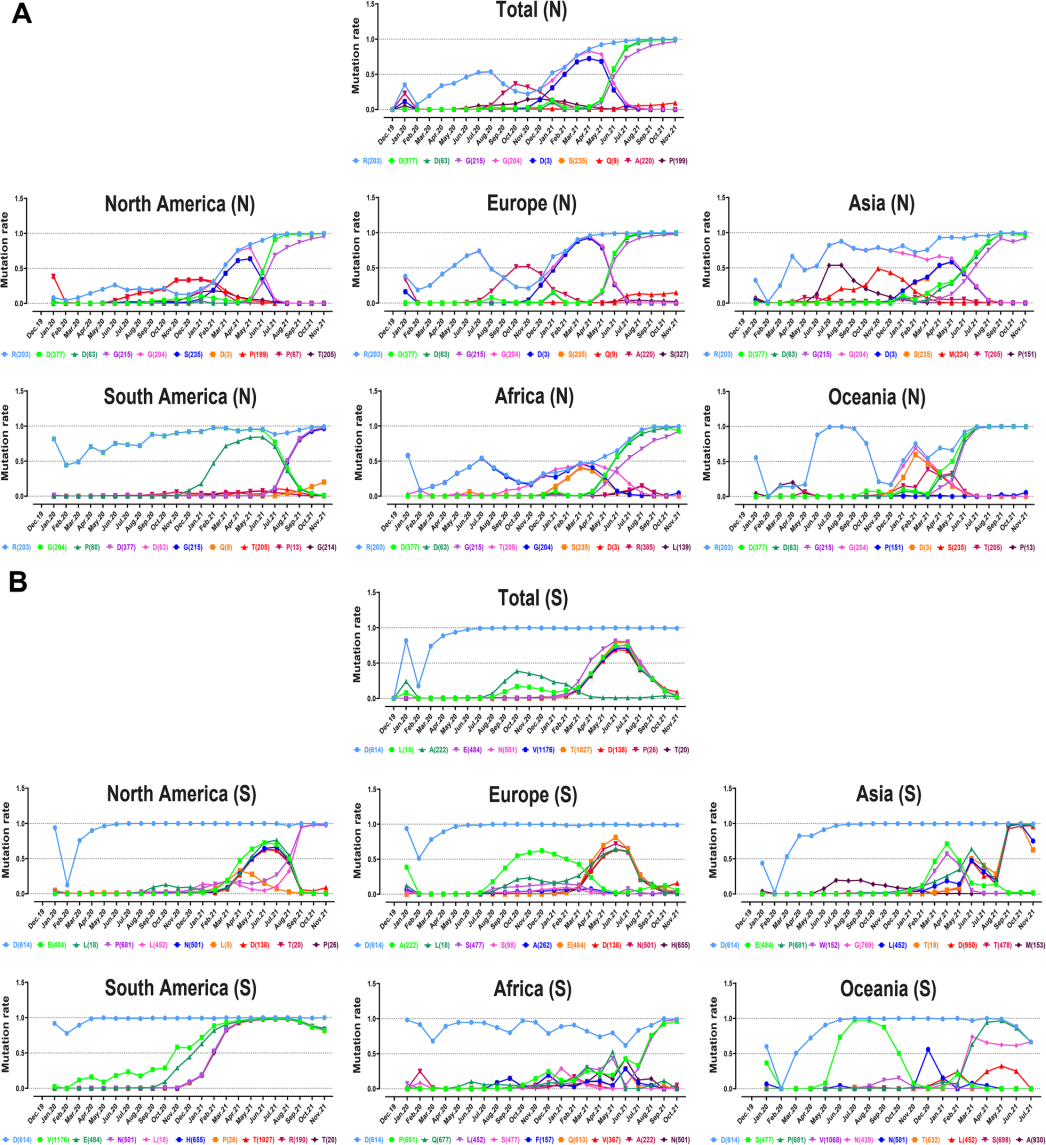
Timeline for reporting the top ten high-rate mutations in the N and S proteins of SARS-CoV-2 across a variety of continents, including North America, South America, Europe, Asia, Oceania, and Africa

Identifying the trends of mutation emergence and spreading can lead to a more practical approach to help identify factors affecting drug and vaccine effectiveness. Figures 9 and 10 display the distribution pattern of the top ten mutations based on collection time, and Additional files 5, 6, 7 and 8 provide supplemental data.

The T9 mutation, which is the most frequent mutation in E AASs in the world (0.0693 frequency rate), began to prevail in October 2021 and till January 2022 (Fig. 9A). P71 mutation prevalence increased in May 2020, then decreased in August 2020, then increased again in September 2020. In March 2021, P71 mutation frequency was 0.0257. Although V62 mutation was present from the beginning of the pandemic, it increased from August and reached maximum frequency in October 2021. (0.0058). P71 emerged in August 2020 in Africa. It then increased till April 2021 and reached the highest frequency (0.3673) and decreased till September 2021. Accordingly, the highest frequency of P71 mutation in all other continents is almost identical to Africa. In Asia, V62 mutation increased noticeably from august 2021 and displayed its maximum frequency rate (0.0901) in October 2021. Since the pandemic began in South America, V58 mutation frequency increased to 0.0457 in May 2020. However, it declined from July 2020.

The most worldwide frequent mutation of M AASs, I82, had a notable frequency rate in January 2021 (0.1095). The second global peak of I82 prevalence started from May 2021 and reached the highest frequency rate (0.9969) in October 2021 (Fig. 9B). From this perspective, except in South America, the evolutionary trends in the distribution of I82 mutation in all continents have almost identical patterns. Although I82 was detectable in South America from the start of the pandemic, it had a near-zero frequency rate before April 2021 and began dominating in July 2021. Q19 mutation increased worldwide in October 2021, when I82 mutation decreased.

The prevalence of mutations among N AASs is fluctuant. R203 mutation has a maximum frequency rate in January 2020and began to increase from February 2020 till august 2020 (Fig. 10A). It began to dominate globally in November 2020 and grew to 0.9907 in January 2022. The evolutionary trends in all areas except South America showed a similar pattern of R203 prevalence. The achieved data from this area demonstrated an almost steady pattern of frequency rate for R203 from April 2020 to January 2022. The evolutionary trends of D63 and G215 mutations have a similar pattern and both started increasing in April 2021 on all continents. Also, P80 mutation, which is common and exclusive in South America, increased from November 2020 to June 2021 and then began to decline.

The growing evolutionary trend of D614G mutation started in February 2020 in the entire world. In contrast to other regions, the mentioned mutation did not follow a consistent pattern in Africa and showed fluctuations. L18 mutation has increased since August 2020 and began to decrease from July 2021 worldwide (Fig. 10B). Such a pattern has also been demonstrated by E488 and N501 globally. Contrary to them, A222 mutation displayed a different trend; from July 2020 to October 2020, its prevalence increased. In May 2020, S477 mutation, one of Oceania’s top ten mutations, increased and decreased 3 months later (August 2020). South American results showed that, except for D614, mutations increased in November 2020.

### Protein–protein interaction (PPI) network presentation

**Fig. 11:**
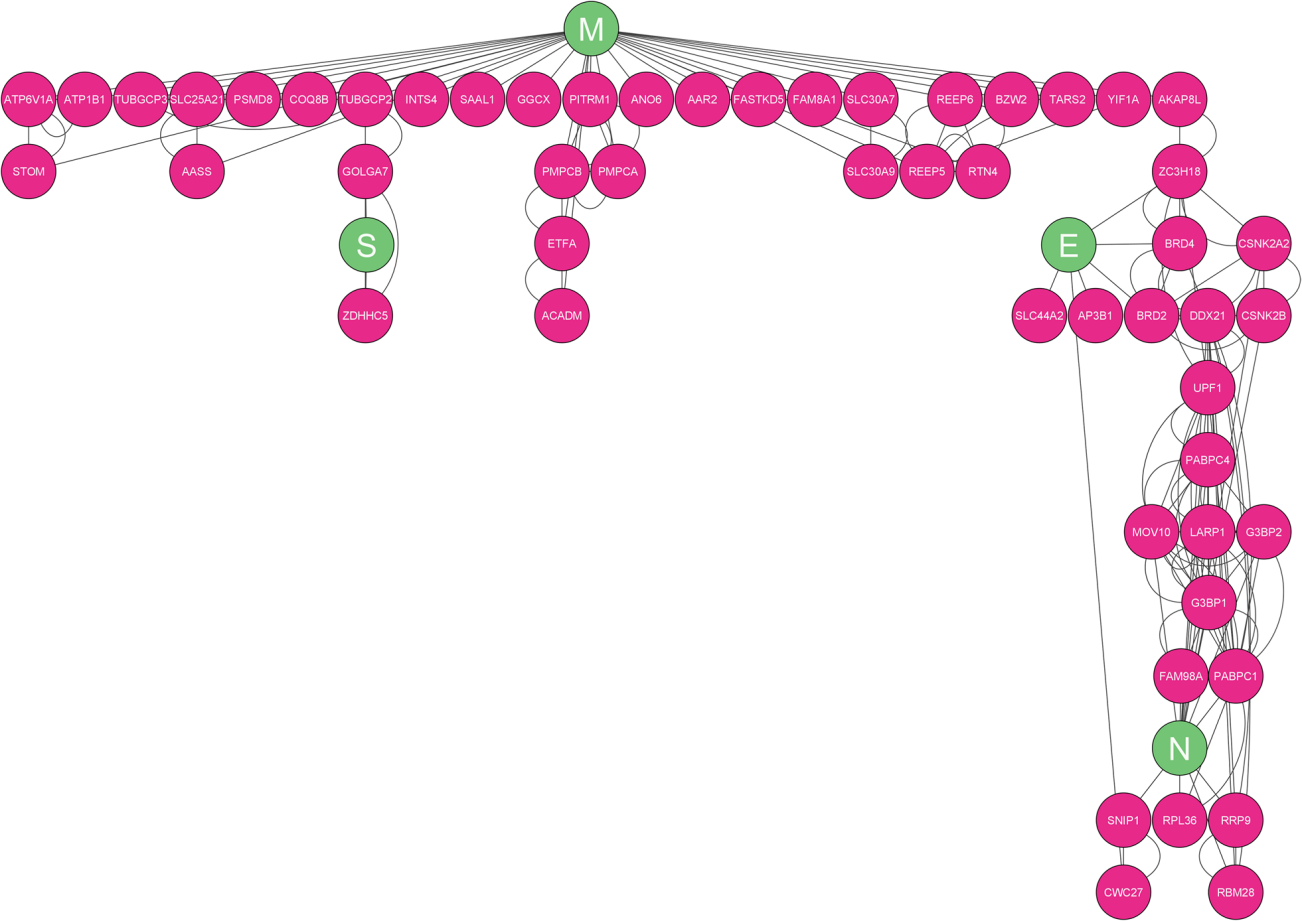
The visualization of the PPI network with 57 nodes and 153 edges

**Fig. 12:**
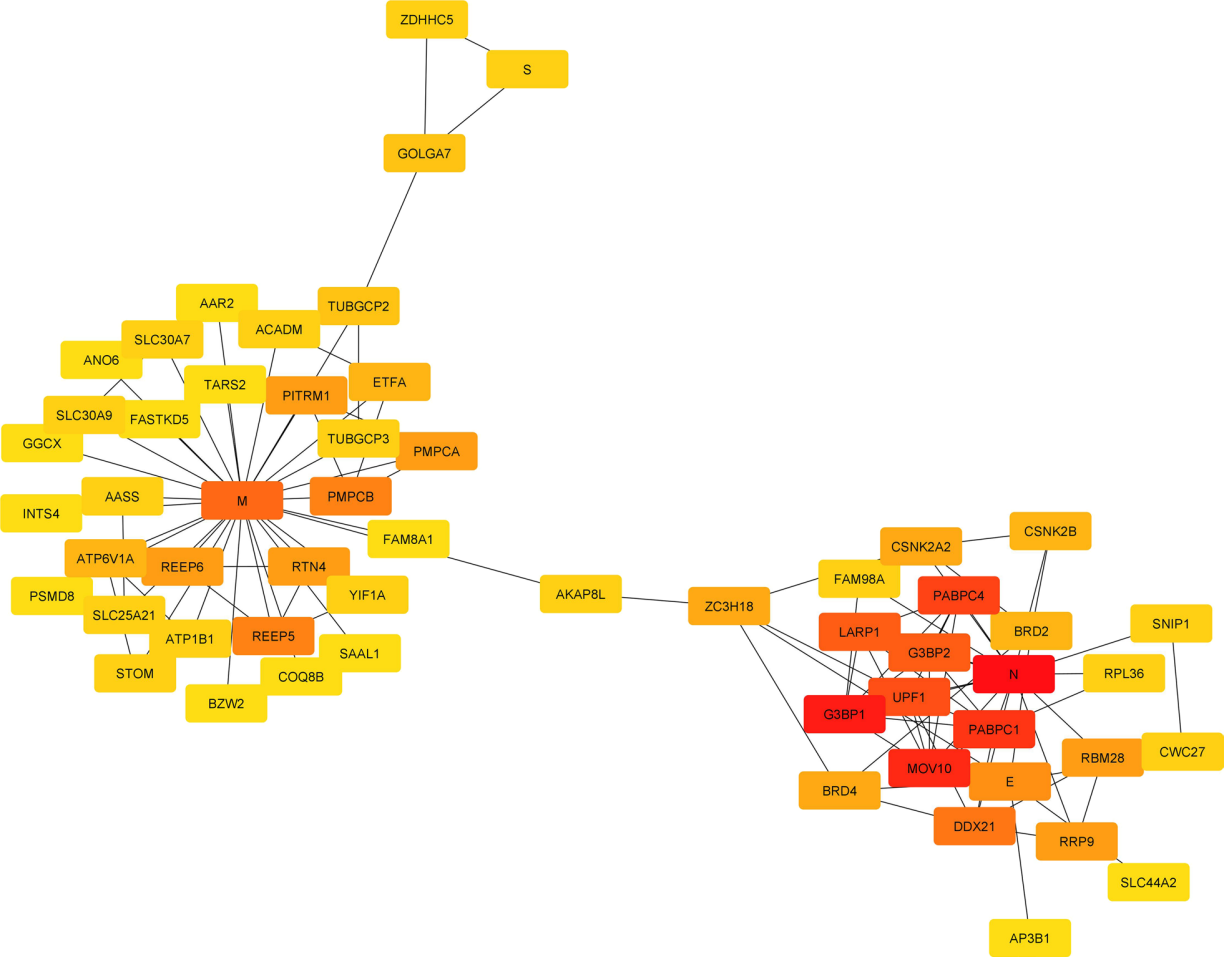
Hub genes identification was concluded by node ranking analysis. As it is seen, the AKAP8L human gene is the linkage gene between the M protein cluster gene and the E and N genes of SARS-CoV-2. Moreover, ZDHHC5 and GOLGA7 showed high interactions with the S protein of SARS-CoV-2.

The protein–protein interaction (PPI) network with 57 nodes and 153 edges presents the interaction between E, M, N, S SARS-CoV-2 protein and human proteins (Fig. 11) (See Additional file 9). Through the ranking analysis, Ras GTPase-activating protein-binding protein 1 (G3BP1) was identified as high human gene rank (Fig. 12). Additional data is illustrated in Additional file 10. The network showed the linkage between the M protein cluster genes and E and N members which are linked with the A-kinase anchoring protein 8 like (AKAP8L) human gene playing a role as a bottleneck. Also, in this network, Zinc Finger DHHC-Type Palmitoyltransferase 5 (ZDHHC5) and Golgin A7 (GOLGA7) have been shown as the human genes with the highest interaction with S protein.

## Discussion

Since March 2020, when COVID-19 was declared a pandemic, it has spread globally with varying results. This variability is due to differences in infectivity and mortality potentials of the dominant virus influenced by community characteristics like age, genetic basis, and mutations [22]. Vaccination and other therapeutic approaches have the potential to assist in putting a stop to the epidemic. Drugs and vaccines that specifically target structural proteins are currently being developed by scientists [17, 23]. As a result, taking into account mutations in these proteins and determining their effects on functions will aid in the high-quality production and development of such preventional and remedial tools.

Focus on the S protein as a pathogenicity-initiator and antibody-stimulator led to identifying new mutations and considering drug and vaccine efficacies for new variants. N501Y is one of 17 mutations in the B.1.1.7 (Alpha) viral genome that enhances viral attachment and infectivity [24]. N501Y appeared in the UK and USA in December 2020, consistent with the evolution of S AA position 501. The mutation increases viral binding affinity and infectivity [25]. Furthermore, the B.1.351 (Beta) variant reported in the second wave of COVID-19 in South Africa in October 2020 had nine mutations, including one at S AA position 501. Accumulation of these mutations, which were among the top ten most common in our study, has been shown to increase transmission and reduce monoclonal antibody neutralization [26]. The B.1.1.28 (Gamma) variant, first detected in Brazil in December 2020, has ten mutations in the S protein: L18F, T20N, P26S, D138Y, R190S, H655Y, T1027I, V1176, K417T, E484K, and N501Y [27]. In the months of March and April 2021 in the state of Paraná, a new mutation called E661D was discovered in the spike (S) protein. This mutation was found in nearly 10% of the genomes that were classified as VOC Gamma [28]. We found that all these mutations were among the top ten most common in South America, except for S AA position 138. Mutations at positions 18, 417, and 484 in the RBD enhance viral binding in Beta and Gamma variants. The next VOC, the Delta variant, caused the April 2021 COVID-19 wave in India, which has six mutations in addition to T19R, T478K, P681R, and D950N, and like Gamma, ten mutations in S protein [29]. Using nanopore sequencing, researchers have also found some mutations in Delta sequences that are not as well characterized in the S protein namely A262S, Q675K, I850L, Q1201H, V1228L, and M1237I, suggesting that they may contribute to the development of future emerging variants [30]. In the current study, the first four mutations were shown to be common in Asia and to have increased after April 2021. T19R, T478K, P681R, and D950N mutations help the Delta variant escape the immune system and increase viral attachment and replication [31, 32]. Prior variants also have a mutation at S protein AA position 681, like the Delta variant, the Alpha variant has P681H mutation. Proline to histidine is the most common mutation at S AA position 681 in North America and the second most common mutation everywhere except Europe and South America. Asia, Africa, and Oceania prefer P681R over P681H. Both mutations increase S1/S2 site cleavage by furin enzyme, they also increase COVID-19 infectivity and virulence [33, 34]. L452R is another RBD of the S protein Delta mutation. According to our study, this mutation is one of the ten most common in Asia, Africa, North America, and Oceania. In agreement with our results, investigating the mutation profile in S AASs among six regions of Asia led to displaying L452R among the most frequent mutations in West Asia, South Asia and Southeast Asia [35]. On the other hand, the most frequent mutations of S AASs did not include L452R in the regions of North Asia, Central Asia, and East Asia. L452R and T478K together can increase viral transmission by affecting viral attachment. E484K/N501Y double mutations in RBD have similar effects in Beta and Gamma [36]. The number of mutations in the Omicron variant, the last VOC, is increasing daily. Because of these mutations, especially in the S protein, it is believed that the end of fatal COVID-19 cases is nearer than before [11, 37]. Our data showed ongoing frequencies of two N protein mutations, G204R and R203K, near the time of variant identification in Africa. During the research period, R203M was more common than R203K on all continents except South America. These two mutations increase viral replication and infectivity [38]. D614G is the most common S protein mutation worldwide and on each continent in which aspartic acid to glycine substitution improves ACE-2 binding leading to increased transmissibility and viral load [3]. Despite these findings, there is no evidence linking D614G to increased COVID-19 severity [3, 8]. Even though the mutation increases antibody neutralization sensitivity, higher infectivity, transmissibility, and lack of effect on severity makes it the most common S protein mutation globally.

M protein is another structural protein that scientists overlook. Despite being highly conserved between SARS-CoV and SARS-CoV-2, its role in viral assembly and pathogenicity is essential [39]. We found that the most common M mutation globally was I82T. Consistently, another study on M mutations in a region found similar mutation frequency results [40]. In November 2021, Q19E mutation in the Omicron variant increased in Africa and globally.

E protein, the smallest structural protein of SARS-CoV-2, is crucial for viral assembly and pathogenesis. Previous studies found that E mutations evolve slowly, as we did [1, 41, 42]. T9I mutation, the most common mutation globally, will increase in November 2021. This is an Omicron mutation. Our study found that E mutations progress more slowly than others globally. 96.40% of E AASs were mutation-free, according to our results. Due to its high conservancy, this structural protein may be a target for COVID-19 vaccine and drug researchers [43].

Other structural proteins can have similar or better drug and vaccination development results than S protein. N protein detection of SARS-CoV-2 is a new serological method [37, 44]. Because the conserved regions in the SARS-CoV-2 N protein have the highest AA similarity with SARS-CoV, researchers should investigate more specific regions to develop new methods based on it [45]. Mutations affect diagnostic test results. Rapid antigen tests can falsely detect T135I mutation. Although this mutation was not among the top ten most common N protein mutations in this study, it is vital to screen mutations accurately to develop reliable diagnostic tests. The mutations, frequencies, and functions of structural proteins could help provide therapeutic strategies. Some S protein mutations, such as E484K and L452R, reduce antibody sensitivity and require higher convalescent sera titers for neutralization [10, 46, 47]. N501Y mutation reduces sensitivity to COVA1-12 and CB6 antibodies but does not affect the neutralizing activity of plasma or sera from vaccinated people [48–50].

Identifying human genes and proteins related to SARS-CoV-2 can expand our knowledge of its host targets. For example, mucins, a group of high-molecular-weight glycoproteins, have been identified as a major viral restriction network that prevents SARS-CoV-2 infection in both cell culture and murine models [51]. According to proteomic studies, ZDHHC5 and GOLGA7 interact heavily with the S protein of SARS CoV-2 [52]. Localized in the ER or Golgi apparatus, ZDHHC5 catalyzes protein palmitoylation. Another cell palmitoylation system that regulates ZDHHC enzyme activity is GOLGA7 [53, 54]. ZDHHCs enzyme and GOLGA7 are essential for palmitoylation of viral proteins, and their high-confidence interaction with SARS-CoV-2 S protein makes them a potential drug target. The current study confirms high ZDHHC5 and GOLGA7 interaction with S protein. G3BP1 was the highest-ranked human gene in our analyses. SARS-CoV-2 N protein interacts with G3BP1 to inhibit host stress granule (SG) assembly and promote viral infection [55]. SGs are dynamic ribonucleoprotein (RNP) assemblies formed during oxidative stress and viral infection. G3BP1 and G3BP2 are key SG-nucleating factors interacting with viral NTF2-like proteins [56, 57]. AKAP8L, the homolog of the scaffold protein AKAP8, links M protein cluster to E and N members in the PPI network, influencing cancer cell tumorigenesis and metastasis [58, 59]. Although the PPI network resulted in the report of related human genes with structural genes of SARS-CoV-2, the exact effects of ZDHHC5 and GOLGA7 on S protein of SARS-CoV-2, the mechanism of interaction between N protein and SG disassembly, and the exact role of AKAP8L in viral infection and its mechanism are unclear. More molecular research is needed to explain the molecular processes.

Two limitations marred our study. First, we studied AASs without nucleotide sequences which prevented checking for codon bias in new variants. Second, different regions report different samples and mutations, leading to ignoring present but undiscovered mutations and not studying new variants.

## Conclusion

Considering the frequencies of mutations affecting virus behavior and their occurrence rate highlights the fact that the rate of vaccination and drug development against COVID-19 is bound to the virus mutations. Therefore, the faster the mutations are screened, the faster development strategies are implemented to combat COVID-19 effectively.

## Supplementary Information

**Additional file 1:** Mutation’s features of the E protein and the attributed frequencies among six continents and globally.

**Additional file 2:** Mutation’s features of the M protein and the attributed frequencies among six continents and globally.

**Additional file 3:** Mutation’s features of the N protein and the attributed frequencies among six continents and globally.

**Additional file 4:** Mutation’s features of the S protein and the attributed frequencies among six continents and globally.

**Additional file 5:** Evolutionary trends of the E protein mutation distribution based on the time and geographical areas.

**Additional file 6:** Evolutionary trends of the M protein mutation distribution based on the time and geographical areas

**Additional file 7:** Evolutionary trends of the N protein mutation distribution based on the time and geographical areas.

**Additional file 8:** Evolutionary trends of the S protein mutation distribution based on the time and geographical areas

**Additional file 9:** PPI network between structural proteins of SARS-CoV-2 and the human proteins

**Additional file 10:** The node ranking analysis of the related human genes with the structural proteins of SARS-CoV-2

## Data Availability

All our study’s steps were performed per the Declaration of Helsinki [60]. We analyzed the SARS-CoV-2 AASs provided in the GISAID database (https://gisaid.org) with the permission and monitoring of Erasmus Medical Center. The datasets supporting the conclusions of this article are included within the article and its additional files.

## Abbreviations

SARS-CoV-2: Severe acute respiratory syndrome coronavirus 2
AASs: Amino-acid sequences
E: Envelope
M: Membrane
N: Nucleocapsid
S: Spike
COVID-19: Coronavirus 2019
AKAP8L: A-kinase Anchoring Protein 8 Like
S/N/C: Substitutions per nucleotide site per cell infection
ORF: Open reading frames
ACE-2: Angiotensin-converting enzyme 2
VOC: Variant of concern
PPI: Protein–protein interaction
G3BP1: GTPase-activating protein-binding protein 1
ZDHHC5: Zinc Finger DHHC-Type Palmitoyltransferase 5
RBD: Receptor-binding domain
ER: Endoplasmic reticulum
SG: Stress granule
RNP: Ribonucleoprotein
GISAID: Global Initiative on Sharing All Influenza Data
